# The complete chloroplast genome of *Pseudognaphalium affine* (D.Don) Anderb. (Asteraceae)

**DOI:** 10.1080/23802359.2021.1993104

**Published:** 2021-10-23

**Authors:** Li Xie, Jiale Zhao, Rong Liu

**Affiliations:** a School of Preclinical Medicine, Chengdu University, Chengdu, China; b Key Laboratory of Resource Biology and Biotechnology in Western China, Northwest University, Xi'an, China

**Keywords:** *Pseudognaphalium affine*, chloroplast genome, Asteraceae, phylogenetic analysis

## Abstract

*Pseudognaphalium affine* (D.Don) Anderb. is an annual herbaceous plant used as a vegetable and traditional medicine. Here, we sequenced and assembled the complete chloroplast genome of *P. affine*. The plastome is 151,573 bp in size with a pair of inverted repeat regions (IRs) of 24,849 bp each, a large single-copy region (LSC) of 83,632 bp, and a small single-copy region (SSC) of 18,243 bp. The overall GC content of the whole plastome is 37.33%, and the IR regions are more GC rich (43.08%) than the LSC (35.30%) and SSC (31.03%) regions. It contains 129 genes, including 83 protein-coding genes, 36 tRNAs, eight rRNAs, and two pseudogenes. Phylogenetic analysis showed that *P. affine* is most closely related to *Leontopodium leiolepis*. This genome will provide a useful genetic resource for future conservation, evolution, and phylogeny studies of *P. affine* and the tribe Inuleae.

*P. affine*, commonly known as Cudweed or Ching Ming vegetable in China, is an annual herbaceous plant that belongs to the tribe Inuleae (Asteraceae). It has long been used as a wild vegetable to process a variety of foods, as well as a traditional Chinese medicine for the treatment of cough, asthma, rheumatic arthritis, and gout (Xi et al. 2012; Zheng et al. 2013). Despite the importance of this species, there have been no genomic studies on *P. affine*, which hampers the understanding of its genomic characterization and evolutionary history. In this study, we reported the first complete chloroplast (cp) genome sequence of *P. affine*.

The fresh leaves of *P. affine* were collected from the Wangjiang Campus of Sichuan University, Chengdu, China (30°38′7″N, 104°5′14″E). The voucher sample is stored in the museum of Chengdu University of TCM (Rong Liu, liurongscu@126.com, accession number: CDU20200906001). Total genomic DNA was extracted with a modified CTAB method (Doyle and Doyle 1987). A paired-end library with an insertion size of 350 bp was prepared and sequenced on an Illumina Hiseq 2500 platform. A total of 5.93 Gb Illumina short reads were generated. Low-quality reads and adaptor sequences were removed. The plastome of *P. affine* was assembled using NOVOPlasty (Dierckxsens et al. 2017) with the plastome of *Anaphalis sinica* (GenBank accession no. NC_034648) as the reference (Lee et al. 2017). The assembled plastome was annotated by using Plann (Huang and Cronk 2015), and the annotation was corrected by using Geneious (Kearse et al. 2012).

The complete cp genome of *P. affine* (GenBank accession no. MW762594) is 151,573 bp in size with a pair of inverted repeat regions (IRs) of 24,849 bp each, a large single-copy region (LSC) of 83,632 bp and a small single-copy region (SSC) of 18,243 bp. The overall GC content of the whole plastome is 37.33%, and the IR regions are more GC rich (43.08%) than the LSC (35.30%) and SSC (31.03%) regions. A total of 129 genes were identified, including 83 protein-coding genes, 36 tRNAs, eight rRNAs, and two pseudogenes (*rps19* and *ycf1*). Among them, six protein-coding genes, seven tRNA genes, and four rRNA genes are duplicated in the IR regions. A total of 18 genes have introns, among which *clp*P and *ycf3* have two introns.

Phylogenetic analysis was performed among *P. affine* and five other species in tribe Inuleae with two species from trib Heliantheae as the outgroup. All of the complete cp genome sequences were aligned by using the Linsi option of MAFFT v7.313 (Katoh and Standley 2013). Then, a maximum likelihood (ML) tree was constructed by RAxML v8.2.11 (Stamatakis 2006) with 100 bootstrap replicates based on the alignments, with the main parameters of ‘-f j -m GTRGAMMA’. The resulted phylogenetic tree showed that *P. affine* is most closely related to *Leontopodium leiolepis* with 100% bootstrap support (Figure 1). The complete cp genome of *P. affine* will provide a useful genetic resource for further studies of this species and the tribe Inuleae.

**Figure 1.**
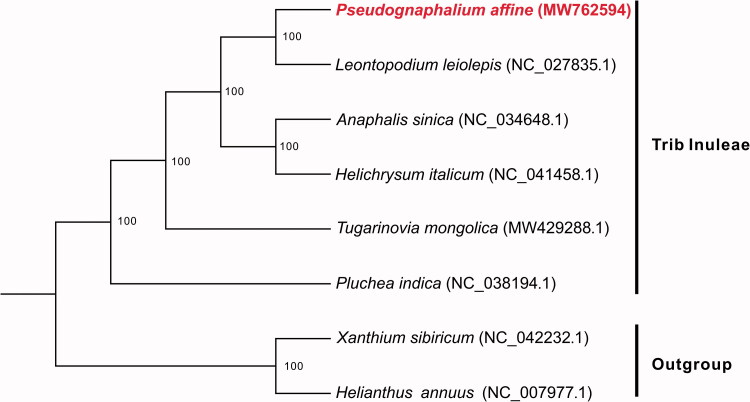
Maximum likelihood phylogeny based on eight complete chloroplast genome sequences, with *Xanthium sibiricum* and *Helianthus annuus* as the outgroup. The number on each node indicates the bootstrap value.

## Data Availability

The chloroplast genome and raw sequencing data in this study are available in NCBI (https://www.ncbi.nlm.nih.gov/) under the accession numbers of MW762594 and SRR13993916 (BioSample: SAMN18344126; BioProject: PRJNA715291).
